# Epstein-Barr viral corneal stromal keratitis occurring during rheumatoid arthritis treatment: a case report

**DOI:** 10.1186/s12886-022-02257-6

**Published:** 2022-01-21

**Authors:** Kazuya Yamashita, Rio Sato, Ryuki Fukumoto, Yoshiko Ofuji, Takashi Nagamoto, Hirohisa Kubono, Mari Kawamura, Kotaro Suzuki

**Affiliations:** Department of Ophthalmology, Keiyu Hospital, 3-7-3 Minatomirai, Yokohama Nishi-ku, Kanagawa 220-8521 Japan

**Keywords:** Epstein-Barr virus, Stromal keratitis, Rheumatoid arthritis, Multiplex polymerase chain reaction, Uveitis

## Abstract

**Background:**

A case of Epstein-Barr viral (EBV) corneal stromal keratitis during rheumatoid arthritis (RA) treatment is presented.

**Case presentation:**

A 74-year-old female undergoing RA treatment was previously treated for bacterial corneal ulcer and herpetic keratitis and healed with antibiotic eye drops and topical anti-herpes ointment. At the first visit to our hospital, she presented with findings of monocular posterior interstitial keratitis with neovascularization mostly located in the inferior cornea with a corneal epithelial defect. The right eye showed no thinning of the corneal periphery and anterior uveitis. Her RA had subsided with oral steroid treatment, and infectious mononucleosis (IM) had not developed. EBV DNA could be detected in her corneal sample. After an extended but ineffective period to antibiotic treatment the corneal infiltrate responded rapidly to topical corticosteroids.

**Conclusion:**

EBV can cause stromal keratitis without IM during treatment for RA.

## Background

Various reports are implicating Epstein-Barr virus (EBV) as the cause of intraocular inflammation. EBV can cause necrotizing retinitis in immunocompromised patients [1, 2]. Interestingly, EBV has been reported to rarely cause keratitis in patients with or without clinically evident infectious mononucleosis (IM) [3–6]. The mechanism by which EBV infects ocular tissue is still unclear, but an immune pathogenesis of EBV-related stromal keratitis is suggested [7]. Herein, we describe a case of EBV-related stromal keratitis without IM undergoing treatment for rheumatoid arthritis (RA).

## Case presentation

A 74-year-old woman presented to our hospital for a nonresolving right corneal ulcer in June 2021. She had been previously treated with antibiotics (moxifloxacin), 1% atropine eyedrops, and acyclovir (ACV) ointment for 4 days before she came to our hospital. Her past ocular history was a right corneal ulcer treated with moxifloxacin eyedrops in 2014, and she was treated for right eye herpes keratitis with moxifloxacin eyedrops and ACV ointment in 2020. Her medical history included current treatment for RA consisting of prednisolone 2 mg and methotrexate 2 mg. She did not use contact lenses. She also denied previous ocular trauma and medical or environmental allergies. Her family history was negative. The best-corrected visual acuity in her right eye since childhood was 20/200 because of anisometropia.

**Fig. 1:**
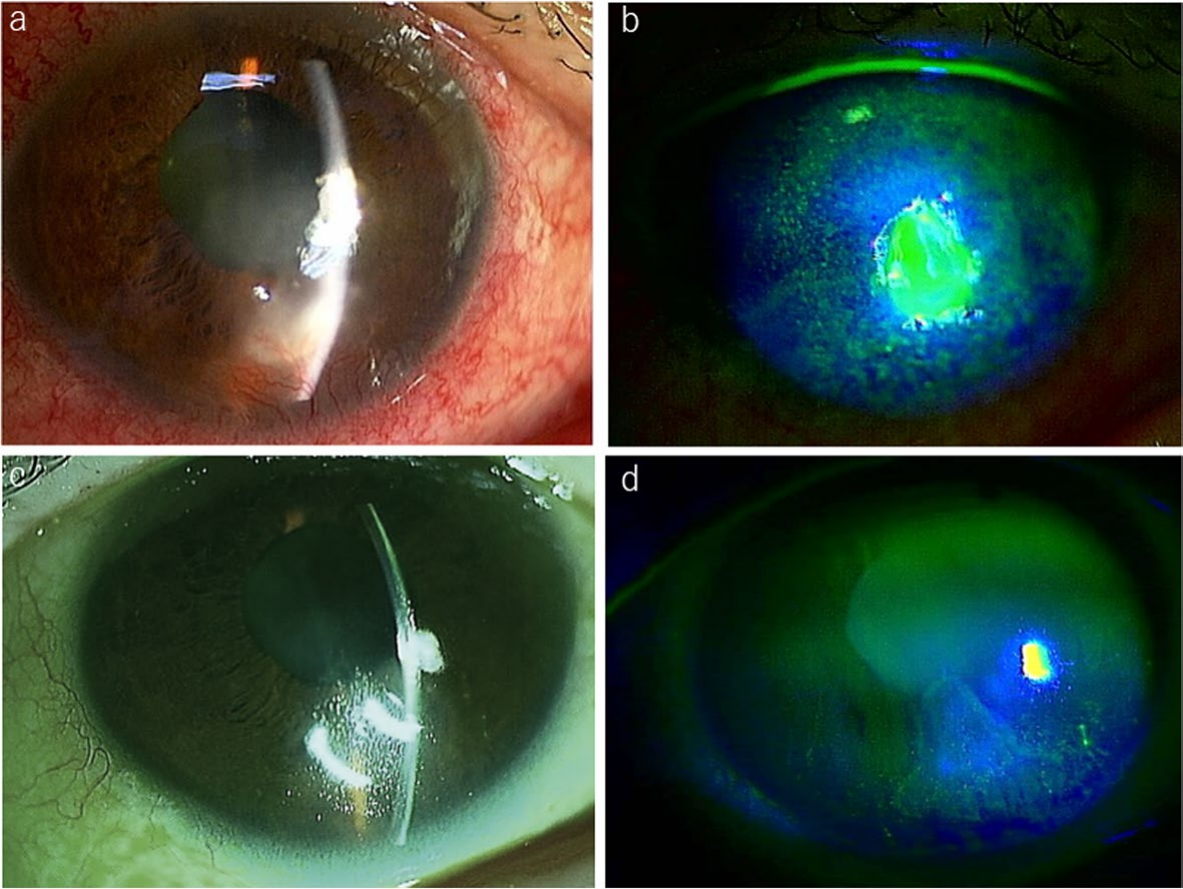
**a** Slit-lamp examination of the right eye at initial presentation showing soft, blotchy, multifocal infiltrates, and thinning of the center area, predominantly involving the peripheral cornea at all depths with neovascularization. **b** Bluelight slit-lamp photograph with fluorescein dye stain showing corneal epithelial defects and an absence of signs of nonthinning or melting of the corneal periphery. **c** Slit-lamp examination in the healing stage showing stabilization of corneal thickness, healing of interstitial keratitis, and development of scar tissue. **d** Bluelight slit-lamp photograph with fluorescein dye stain in the healing stage showing disappearance of the corneal epithelial defects, slight superficial punctate keratopathy

**Fig. 2:**
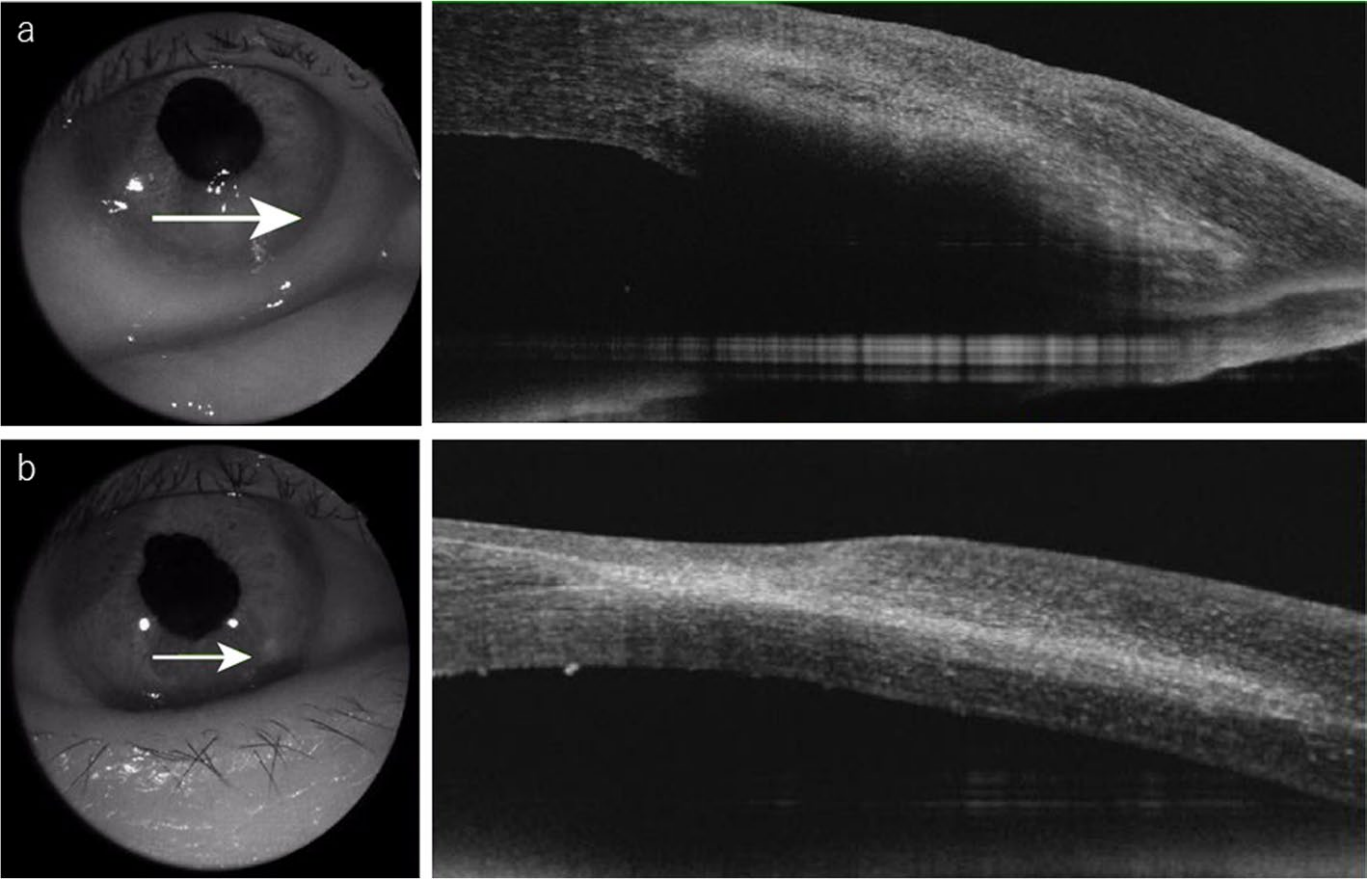
**a** Anterior segment OCT findings at initial presentation showing an irregular surface luminance of the stromal border, unusual reflectivity of the stroma, and swelling towards the corneal endothelium, but the corneal endothelium was intact. **b** Anterior segment OCT findings in the healing stage showing a thick intact epithelium, hyporeflectivity and decreasing thickness of the stroma

**Fig. 3:**
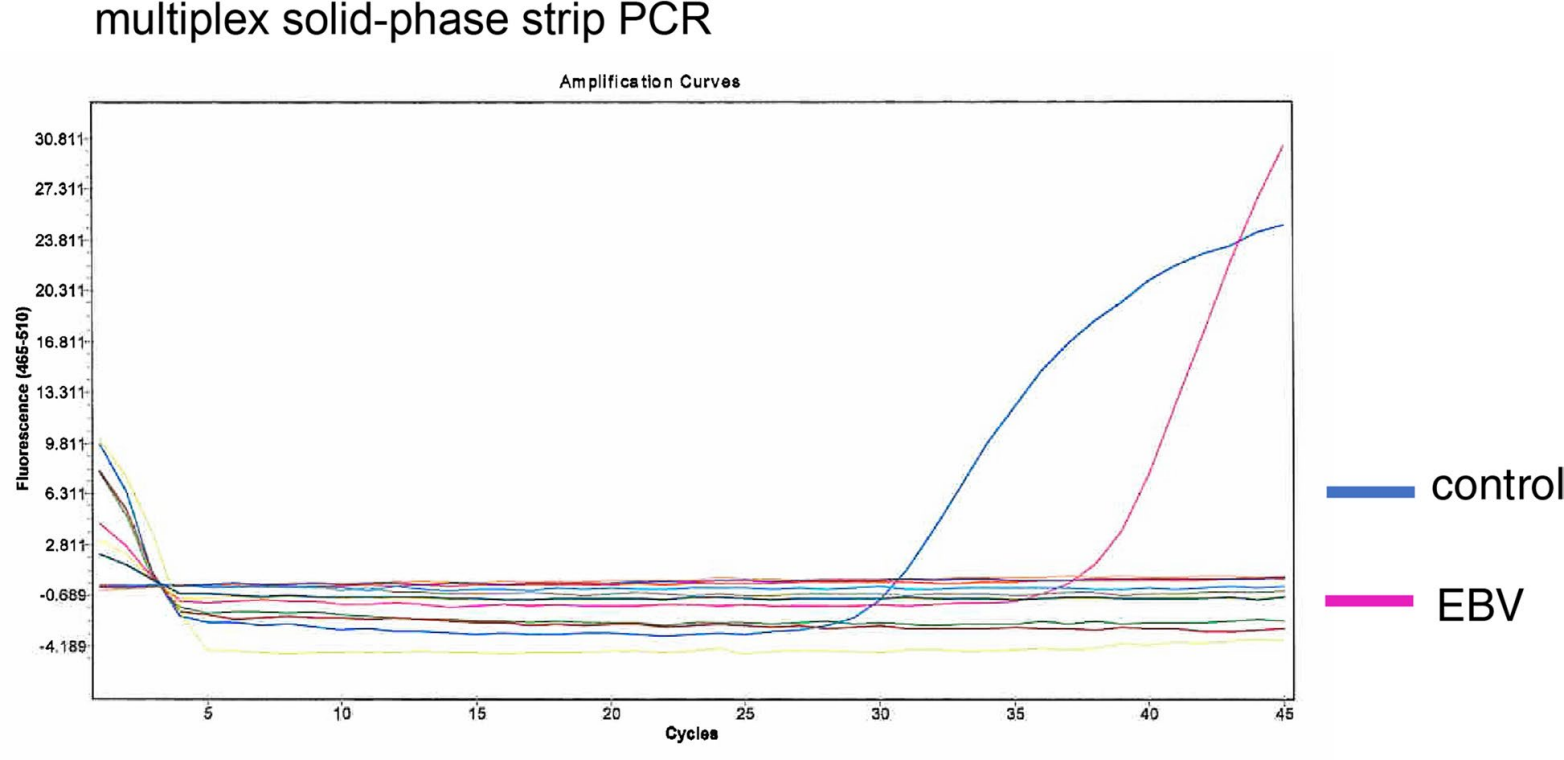
Representative multiplex solid-phase strip PCR results of a cornea sample: The sample is positive for EBV-DNA, with 6.86 × 10E-1 copies/μg DNA

At presentation, the patient’s main complaint was pain, redness, irritation, and photophobia. She denied general complaints such as an autoinflammatory condition, consistent with periodic fever, aphthosis stomatitis, pharyngitis, adenitis (PFAPA) syndrome over the past 12 months. Corneal sensation was intact. No preauricular or submandibular lymphadenopathy was present. She had a best corrected visual acuity of 20/250 in the right eye and 20/17 in the left eye. Slit-lamp examination showed a corneal epithelial defect and distinct inferior interstitial keratitis with neovascularization affecting the deeper layers of the stroma and extending directly from the limbus without keratic precipitates. The internal growth of the blood vessel reached about 3 mm anterior to the corneal limbus with active pannus extending from 5 o’clock to 6 o’clock (Fig. 1 a, b). Anterior segment optical coherence tomography (DRT OCT Triton Plus, TOPCON, Tokyo, Japan) showed an irregular surface luminance of the stromal border, unusual reflectivity of the stroma, and swelling towards the corneal endothelium, but the corneal endothelium was intact (Fig. 2 a). No signs of anterior uveitis were noted, and the laser flare meter (FM-600, KOWA Co., Ltd., Aichi, Japan) showed a value of 19.3 ± 1.4. No abnormalities were detected in the posterior segment. The corneal lesions were cultured for bacteria, including Chlamydia, and viruses concomitantly. The DNA of the right scratched corneal sample and of the right aqueous humour were each separately extracted using a DNA Mini kit (Qiagen, Valencia, CA). The DNA was then processed for multiplex solid-phase strip PCR testing targeting 24 specific genomic sequences of human herpesviruses and other pathogens, e.g., herpes simplex virus (HSV) 1, HSV2, varicella-zoster virus (VZV), Epstein-Barr virus (EBV), cytomegalovirus (CMV), human herpes virus (HHV) 6, HHV7, HHV8, adenovirus, human T-cell lymphotropic virus (HTLV)-1, *Treponema pallidum*, *Mycobacterium tuberculosis*, bacterial 16S ribosomal RNA (rRNA), *Propionibacterium acnes* (*P. acnes*), *C. glabrata*, Candida species (Candida sp.), Aspergillus, *C. krusei*, fungal 28S rRNA, Fusarium, Toxocara, Toxoplasma (T. gondii), Acanthamoeba and *Chlamydia trachomatis* (*C. trachomatis*) [8–12]. Real-time PCR for the positive pathogens in multiplex solid-phase strip PCR was also performed. Multiplex solid-phase strip PCR and real-time PCR were performed using a LightCycler 480 II instrument (Roche, Basel, Switzerland). The primers, probes, and PCR conditions used for the above pathogens have been described previously [8–12]. The results showed that only EBV-DNA was detected with a load of 6.86 × 10E-1 copies/μg in the corneal sample, both PCR-exams (multiplex solid-state PCR and real-time PCR) were positive and we were able to exclude the other 23 pathogens, such as HSV1 and Acanthamoeba (Fig. 3). Extensive testing was conducted to rule out infectious and autoimmune causes of interstitial keratitis (serum IgM and IgG for EBV, TPA and ACE; X-ray of the mediastinum and chest). Only EBV serology was found to be positive, displaying a panel compatible without IM (VCA IgM negative, VCA IgA negative, VCA IgG 1:160, EB-nuclear antigen (EBNA) negative).

The patient was started on topical antibiotics (tobramycin, cefmenoxime, and moxifloxacin eye drops) after discontinuing topical treatment, including ACV ointment, prescribed by a previous doctor. After confirming that bacterial culture was negative and EBV-DNA was detected in the corneal stroma sample, we started her on topical steroid eye drops. The clinical picture at 1 week of treatment showed better resolution, epithelial staining was reduced, and the inferior pannus showed significant resolution, with regression of the vessels. Two weeks later, the patient’s symptoms improved. The vessels had remarkably reduced and the only corneal haze was in the area of the previous vascular pannus (Fig. 1 c, d). She showed no PFAPA (periodic fever, aphthosis stomatitis, pharyngitis, adenitis) syndrome symptoms and no signs of diseases in the left eye during the course. Anterior segment OCT revealed a thick intact epithelium, hyporeflectivity, and decreasing thickness of the stroma (Fig. 2 b).

## Discussion and conclusions

Our case presented stromal keratitis with neovascularization, primarily affecting the inferior peripheral cornea. She had RA and was undergoing treatment with prednisolone and methotrexate. The patient did not have a history of previous IM, she showed no EBV activity such as PFAPA syndrome symptoms during the course. Given her clinical course, the second serum test considering the window period was not performed. Interestingly, no iritis was present, and EBV DNA could be detected in the corneal sample. The occurrence of EBV-related stromal keratitis is uncommon; however, a few cases have been reported to date [7, 13, 14]. To the best of our knowledge, this is the first report of a case of stromal keratitis without IM undergoing treatment for RA.

In previous reports, only one case of nummular keratitis with endothelial precipitates showed the presence of EBV DNA in the aqueous humour of the patient [14]. Chodosh et al. [15] reported the presence of EBV DNA in the aqueous humour of 5 out of 10 cadaveric healthy eyes, and only three eyes had EBV DNA present in the cornea. EBV DNA was not detected in 110 corneas excised for penetrating keratoplasty [16]. Previous reports showed poor correspondence between corneal and aqueous humour EBV DNA positivity.

The mechanism by which EBV causes keratitis is presently unknown. EBV-related stromal keratitis appears to manifest months after IM, and the delayed presentation in conjunction with the limbal location and the lack of anterior uveitis would suggest an immune pathogenesis [7]. In humans, after the initial infection, EBV persists in a latent form in memory B-lymphocytes, and the immortalization of B-cells associated with the ability to elicit a strong T-cell response creates a very favourable environment for the development of autoimmunity [17]. In addition, several EBV antigens have been implicated to have mechanisms associated with cross-reactivity and antigen mimicry [18]. We thought EBV might be latently infected in the stroma of the cornea, the presence of RA caused EBV reactivation.

EBV has long been suspected to be associated with the pathophysiology of RA [18]. The presence of EBV DNA/RNA has been demonstrated in RA patients [19–23], and RA patients have 10-fold higher frequencies of EBV-infected B-cells than in healthy controls [24]. Furthermore, previous reports have shown a humoural response to both latent and lytic EBV antigens with elevated titres of antibodies against EBNA1, VCA, and EA/R in both the serum and synovial fluids from RA patients higher than in healthy controls [19, 25–27]. A recent study of EBV-specific T-cells in the peripheral blood of RA patients has revealed a defective IFNγ response to EBV proteins compared to healthy controls [28]. Thus, the increased viral load, high titres of EBV-directed antibodies, and decreased cell-mediated control of EBV in RA patients compared to healthy controls may play a role in the infiltration of EBV in the stromal keratitis of RA patients.

The response to corticosteroid treatment in our patient was complete resolution. In previous literature, topical corticosteroid treatment usually induced a brisk and complete resolution of clinical signs and symptoms [13]. What was important in our case was that it was associated with corneal epithelial defects and needed to be differentiated from corneal ulcers. We first stopped the treatment including ACV from the previous doctor in our case for appropriate diagnosis. Although the treatment with antibiotics was prolonged, the detection of EBV-DNA from the corneal sample led to the decision to use topical steroids. Even more interesting is the cure of EBV-related stromal keratitis with antibiotics and steroids. In HSV keratitis, topical ACV combined with a topical steroid therapeutic regimen is known to be highly effective. Topical antiviral drugs stop virus replication, whereas topical steroids limit the corneal immune response and immune destruction [29]. However, a previous meta-analysis with acute IM treated with ACV does not support the use of ACV for the treatment of acute IM, despite the good virological activity of this drug [30], topical and oral antivirals such as ACV were also employed as additional treatments in other series, but the benefit of such treatments is unknown [13, 14, 31]. It is unclear whether this combination therapy of ACV and corticosteroid is effective for EBV-associated keratitis or whether topical steroids alone have the same results as the combination therapy.

In conclusion, we describe a case of stromal keratitis during treatment for RA. Analysis of the DNA from corneal samples may be important when considering the source of the corneal ulcer. Further studies are needed to better delineate the clinical features and to shed light on the pathogenesis of the EBV IM-associated corneal effects.

## Data Availability

All the data supporting the conclusions of this article are included in the present article.

## Abbreviations

EBV: Epstein-Barr virus
RA: Rheumatoid arthritis,
IM: Infectious mononucleosis
OCT: Optical coherence tomography
HSV: Herpes simplex virus
VZV: Varicella-zoster virus
CMV: Cytomegalovirus
HHV: Human herpes virus
HTLV: Human T-cell lymphotropic virus
EBNA: EB-nuclear antigen
PCR: Polymerase chain reaction
PFAPA: Periodic fever, aphthosis stomatitis, pharyngitis, adenitis
ACV: Acyclovir
